# Patients With Infantile Nephropathic Cystinosis in Germany and Austria: A Retrospective Cohort Study

**DOI:** 10.3389/fmed.2022.864554

**Published:** 2022-04-25

**Authors:** Nina O'Connell, Jun Oh, Klaus Arbeiter, Anja Büscher, Dieter Haffner, Jessica Kaufeld, Christine Kurschat, Christoph Mache, Dominik Müller, Ludwig Patzer, Lutz T. Weber, Burkhard Tönshoff, Marcus Weitz, Katharina Hohenfellner, Lars Pape

**Affiliations:** ^1^Department of Pediatric Kidney, Liver and Metabolic Diseases, Hannover Medical School, Hannover, Germany; ^2^Department of Pediatric Nephrology, University Hospital Hamburg-Eppendorf, Hamburg, Germany; ^3^Division of Pediatric Nephrology and Gastroenterology, Department of Pediatrics and Adolescent Medicine, Medical University of Vienna, Vienna, Austria; ^4^Department of Pediatrics II, University Hospital of Essen, University of Duisburg-Essen, Essen, Germany; ^5^Department of Nephrology and Hypertension, Medical School of Hannover, Hannover, Germany; ^6^Department II of Internal Medicine, Center for Molecular Medicine Cologne and Center for Rare Diseases Cologne, Faculty of Medicine and University Hospital Cologne, University of Cologne, Cologne, Germany; ^7^Children's and Adolescents' University Hospital, University of Graz, Graz, Austria; ^8^Division of Pediatric Nephrology, Charité University Medicine, Berlin, Germany; ^9^Children's and Adolescents' Hospital, Elisabeth Krankenhaus, Halle, Germany; ^10^Department of Pediatric Nephrology, Children's and Adolescents' Hospital, University Hospital of Cologne, Faculty of Medicine, University of Cologne, Cologne, Germany; ^11^Department of Pediatrics I, University Children's Hospital Heidelberg, Heidelberg, Germany; ^12^Children's and Adolescents' University Hospital, Universtiy of Tübingen, Tübingen, Germany; ^13^Children's Hospital, Klinikum Rosenheim, Rosenheim, Germany

**Keywords:** cystinosis, dialysis, kidney transplantation, growth, metabolic disease, rare disease, chronic kidney disease

## Abstract

**Background:**

Infantile nephropathic cystinosis (INC) is a rare lysosomal storage disorder resulting in progressive chronic kidney disease (CKD) and a variety of extrarenal manifestations. This orphan disease remains a challenge for patients, their families and health care providers. There is currently no comprehensive study on patients' clinical course in Germany and Austria.

**Methods:**

A retrospective cohort study including 74 patients at eleven centers of care was conducted. Data on time of diagnosis, CKD stage, leukocyte cystine levels (LCL), extrarenal manifestations, and treatment was collected from medical charts and subsequently analyzed using explorative statistics. Age at initiation of kidney replacement therapy (KRT) was evaluated by Kaplan–Meier analyses for different groups of patients.

**Results:**

Patients were diagnosed at a median age of 15 months (IQR: 10–29, range: 0–110), more recent year of birth was not associated with earlier diagnosis. Oral cystine-depleting therapy (i.e., cysteamine) was prescribed at a median dose of 1.26 g/m^2^ per day (IQR: 1.03–1.48, range: 0.22–1.99). 69.2% of all 198 LCL measurements of 67 patients were within the desired target range (≤ 1 nmol cystine/mg protein). Median time-averaged LCLs per patient (*n* = 65) amounted to 0.57 nmol cystine/mg protein (IQR: 0.33–0.98, range: 0.07–3.13) when considering only values at least 1 year after initiation of therapy. The overall median height of 242 measurements of 68 patients was at the 7^th^ percentile (IQR: 1–25, range: 1–99). 40.5% of the values were ≤ the 3^rd^ percentile. Patient sex and year of birth were not associated with age at initiation of KRT, but patients diagnosed before the age of 18 months required KRT significantly later than those patients diagnosed at the age of ≥ 18 months (*p* = 0.033): median renal survival was 21 years (95% CI: 16, -) vs. 13 years (95% CI, 10, -), respectively.

**Conclusion:**

Early diagnosis and initiation of cystine depleting therapy is important for renal survival in children with INC. Cysteamine doses and LCL showed that treatment in this cohort met international standards although there is great interindividual variety. Patient growth and other aspects of the disease should be managed more effectively in the future.

## 1. Introduction

Infantile nephropathic cystinosis (INC), MIM 219800, is a lysosomal storage disorder due to loss of function of the proton/cystine symporter cystinosin on lysosomal membranes. Accounting for 95% of all cases, it is the most common but also the most severe form of cystinosis (1).

It is due to bi-allelic mutations of the 12-exon *CTNS* gene on chromosome region 17p13 (2, 3). Up to now, over 140 different types of mutations have been described, the most common one in Northern Europeans being the 57kb-deletion of the promotor region and the first 10 exons of the *CTNS* gene together with two upstream genes (4, 5).

In Northern European studies conducted during the twentieth century, the prevalence of INC was estimated as 0.3 to 0.9 per 100,000 live births (6). In 2018/19, a screening study of 292,000 German newborns which used multiplex PCR and next-generation sequencing detected 2 patients (7, 8). According to the Interdisciplinary Cystinosis Clinic in Rosenheim, there are currently approximately 130 pediatric and adult INC patients in Germany. At this clinic, all German INC patients have the opportunity to receive standardized and coordinated interdisciplinary care at yearly intervals (9).

In INC, several mechanisms account for the phenotype (10). However, despite many efforts, it is still not fully known how these mechanisms are interlinked or how they impact upon the clinical course of the disease.

Lysosomal efflux of cystine into the cytoplasm is mostly cystinosin-dependent. Following loss of function of cystinosin in INC, the amino-acid accumulates and crystallizes inside the lysosome (11). Alterations in mitochondrial metabolism, lysosomal dynamics, autophagy, apoptosis, the mTORC1-pathway and inflammatory responses have also been explored in INC (10).

A diagnosis of INC is confirmed by measurement of elevated LCL (levels of healthy subjects are below 0.2 nmol cystine/mg protein), slit lamp examination of corneal cystine crystals which become apparent by the age of 18 months at the latest and *CTNS* gene sequencing (1, 12).

Although cystinosin is expressed in all tissues, manifestations of INC vary in degree of severity and time of onset: Children mostly present with renal Fanconi syndrome before their first birthday, which manifests as failure to thrive, polyuria, polydipsia, vomiting, dehydration, electrolyte imbalance, and hypophosphatemic rickets. These symptoms are caused by the failure of renal proximal tubular cells to reabsorb water, glucose, amino acids, uric acid, carnitine, phosphate, bicarbonate, and other small molecules (1). In later life, glomerular function also deteriorates, a phenomenon which has been linked to pathognomonic podocyte injury (13, 14). When treated adequately, the need for kidney replacement therapy (KRT) can be delayed until the second or even third decade of life (15), historical cohorts reached end stage kidney failure at approximately 10 years of age (16).

There are many extrarenal complications which accompany INC and strongly impact a patient's quality of life: Ophthalmological manifestations can affect every part of the eye—an early and frequent symptom is photophobia due to corneal crystal deposition (17). Gastrointestinal symptoms of INC are caused by cysteamine treatment and/or by cystine deposition in the gut wall (18). The most common endocrinological complications of the disease are hypothyroidism, diabetes mellitus (1), pubertal delay (19, 20), and male infertility (21). Muscle impairment is not only of concern for bone metabolism and strength, but may lead to swallowing difficulties and respiratory insufficiency (16, 18). Short stature, skeletal pain and deformities, as well as a higher risk of fractures are more common in INC and growth also seems to follow a different pattern than in other nosologic entities of chronic kidney disease (CKD) (22, 23). A primary defect in cystinotic osteoblasts and osteoclasts as well as secondary implications of INC account for the so-called cystinosis-associated metabolic bone disease (22, 24, 25). Varying individually, neurological (26), hematological, dermatological, cardiac, and psychosocial implications of the disease have also been reported (27).

If left untreated, morbidity and mortality in INC are high (1, 16, 28)—due to the development of adequate specific and supporting therapy, patients with the former solely pediatric disease today might live past the age of 40 years (29): In end-stage kidney disease, dialysis and kidney transplantation are provided. Extra-renal manifestations of INC are treated according to the symptoms, e.g. through administration of thyroxine or growth hormones (12). The only currently available specific therapy for INC is the cystine-depleting agent cysteamine, which is administered orally. Although cysteamine is not a rescue treatment for Fanconi syndrome—and cannot cure INC—it can delay progression of the disease (16, 30). At present, an immediate-release cysteamine bitartrate (IRC, to be administered strictly every 6 h) and an extended-release formulation (ERC, for twice-daily dosing) are available (31). Topical cysteamine application is needed to address the avascular cornea (32).

Improved cysteamine formulations and new approaches based on recently discovered mechanisms are being investigated (10). A Phase I/II clinical trial is ongoing (NCT03897361) to examine safety and efficacy of autologous transplantation of hematopoietic stem cells expressing the *CTNS* gene after lentiviral modification *ex vivo*.

The present study assesses the clinical course of INC in a large German-Austrian cohort, characterizing factors associated with the progression of the disease and exploring possibilities for improvement in the quality and structure of health care.

## 2. Methods

To participate, patients had to be diagnosed with INC and to have received treatment in Germany or Austria within the last 10 years. Written informed consent from the patients themselves and/or their parents/guardians was mandatory. A superordinate ethics committee approval was received by the ethics committee of Hannover Medical School. The study was conducted in accordance with the Declaration of Helsinki.

Baseline data included date of birth, sex, time of initial diagnosis and initiation of systemic cystine-depleting therapy, dialysis and kidney transplantation.

Data was collected retrospectively from the patients' health records in the cooperating hospitals: These included the most recent routine examination along with examination results at approximately 12-monthly intervals from that point until up to 10 years earlier and at the time of the initial diagnosis.

Patients' records on routine examinations were searched for anthropometric data, laboratory parameters, medication and clinical symptoms. Percentiles were calculated for patient height and weight (33). Laboratory parameters to calculate estimated glomerular filtration rate (eGFR) according to Schwartz 2009 [0.413 x (height in cm/serum-creatinine in mg/dl)] (34) and CKD stage according to KDIGO 2012 (35) were documented, as well as the cystine level in mixed white blood cells (nmol cystine/mg protein) to monitor therapy with cysteamine. A leucocyte fraction was isolated *via* density gradient centrifugation. Cystine content was analyzed *via* GC-MS and normalized to protein content, our results were multiplied by a factor of 2 to get 1/2 cystine-results. Cut-off values were 0.1–0.5 nmol cystine/mg protein for carriers and sufficient therapy control in INC patients was defined as LCL below 1.00 nmol cystine/mg protein.

Additional data included information on medication: cysteamine in g/m^2^ per day calculated according to DuBois (36) with overdosing defined at the recommended maximum of 1.95 g/m^2^ per day (12), type of cysteamine (IRC vs. ERC), use of growth hormone therapy and further supporting medication. Manifestation of anemia, hypothyroidism, rickets, skeletal deformities, gastrointestinal, ophthalmological, neurological, and muscular symptoms, as well as the need for tube feeding, were recorded as binary variables.

For statistical analysis, R (version: 4.0.5) was used (37). All documented values for laboratory parameters and cysteamine dosage were included. Continuous data was reported using arithmetic mean and standard deviation (SD) or median, interquartile range (IQR), and range when data did not follow a normal distribution according to the Shapiro–Wilk test. Categorical data was expressed using counts and percentages; relative frequencies always referred to the total number of patients regardless of missing values. The main focus was on Kaplan–Meier-estimators including 95%-confidence intervals (95% CI) and log-rank tests for *p*-value. The level of significance was set at *p* = 0.05. Time until KRT was analyzed (age in years at first dialysis or kidney transplantation) and stratified for age at initial diagnosis, sex and year of birth to investigate whether treatment was related to era. To examine possible correlations of data which did not follow normal distribution, scatterplots, and Spearman's correlation were used. The nearer the absolute value of Spearman's coefficient ρ is to 1, the more the data correlates. Here, *p* was set to 0.02 to increase the probability of rejecting the null hypothesis.

## 3. Results

### 3.1. Study Population

A total of 74 patients from eleven healthcare centers was included in this cohort study. Thirty-nine (52.7%) of the participants were male, 35 (47.3%) were female. Half of the patients were born until 2002 (IQR: 1995–2009, range: 1975–2019). Health records were available for the years 1986 to 2020 (median: 2016, IQR: 2013–2016). Median survey duration was 2 years (IQR: 1–5, range: 1–17). The median age patients had at the recorded examinations was 11 years (IQR: 6–15, range: 0–42). The study cohort mostly represented underage patients at a total of 240 points in time; only 36 observations were made for patients at the age of 18 years and older.

### 3.2. Initial Diagnosis and Initiation of Cystine-Depleting Therapy

Median age at diagnosis of INC (*n* = 67) was 15 months (IQR: 10–29, range: 0–110). In one case the date of initial diagnosis was unknown, while in six cases only the year was given. Spearman's correlation of birth year and age at diagnosis, as well as a scatterplot, showed no correlation (ρ = −0.17, *p* = 0.16).

Systemic cystine-depleting therapy was initiated at a median age of 16.5 months (IQR: 11–30, range: 0–111). Data was missing on 12 patients born between 1975 and 2003. In 53 patients, cysteamine therapy started within 1 month after initial diagnosis, in seven cases within 4 months, whilst two patients (both born in 1981) started treatment 3 and 7 years after initial diagnosis.

### 3.3. Systemic Cysteamine Therapy and Monitoring

For 72 patients, the treatment with systemic cysteamine was documented, data was missing for two patients. The use of IRC was recorded in 54 cases, with 17 patients changing to ERC at a median age of 11 years (IQR: 6–14, range: 2–16). Two patients changed back to IRC after 1 and 2 years, one of them due to increased vomiting. The use of ERC alone was reported for two patients, whilst two more patients took IRC twice during the daytime and ERC once at night-time.

The exact cysteamine dosage was available for 67 patients, with the median of the 235 documented values being 1.26 g/m^2^ per day (IQR: 1.03–1.48, range: 0.22–1.99). 84 values of 35 patients were >1.35 g/m^2^ per day with a median of 1.57 (IQR: 1.46–1.67, range: 1.36–1.99) at a median age of 12 years (IQR: 7–16, range: 1–35). Two patients received a dose slightly above the recommended maximum of 1.95 g/m^2^ per day, with 1.99 each at the age of 12 and 14 years. There was no documentation on side effects available.

The median dose of 171 values of the patients on IRC was 1.31 (IQR: 1.16–1.51, range: 0.22–1.99). The median dose of 71 values of the patients on ERC was 1.03 (IQR: 0.95–1.21, range: 0.27–1.86).

The median time-averaged LCL values based on 65 patients amounted to 0.57 (IQR: 0.33–0.98, range: 0.07–3.13). Here, only measurements at least 1 year after initiation of therapy were considered. 69.2% of all 198 LCL measurements of 67 patients were within the desired target range ≤ 1 nmol cystine/mg protein. An overview of all documented LCLs is given in Table 1.

**Table 1:** Leukocyte cystine levels of 67 patients.

**LCL**		**Median**	**IQR**	**Range**	***n***
≤ 1 (sufficient)	LCL*	0.49	0.28–0.70	0.04–1.00	137 values of 62 patients
	Age	11	7–15	1–42	
≤ 0.5 (optimal)	LCL	0.30	0.20–0.40	0.04–0.50	79 values of 51 patients
	Age	13	7–16	1–42	
>1 (insufficient)	LCL	2.00	1.49–2.70	1.03–19.10	61 values of 27 patients
	Age	7	3–12	1–39	

* *LCL in nmol cystine/mg protein, age in years*.

There was no association between cysteamine dosage and age (ρ = 0.06, *p* = 0.38) or LCLs (ρ = (−0.06), *p* = 0.44).

### 3.4. Estimated Glomerular Filtration Rate

**Figure 1:**
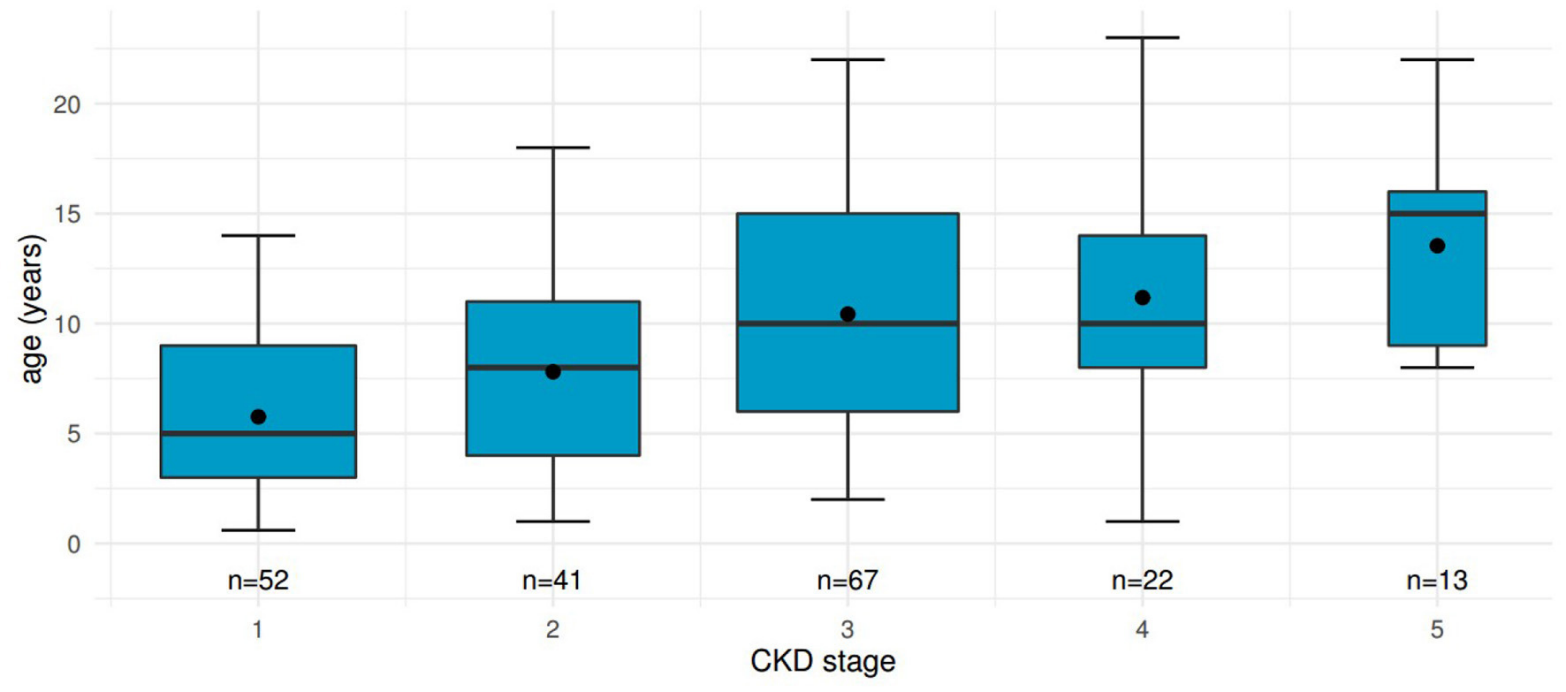
Distribution of age at different CKD stages in patients before kidney transplantation. Boxplots including mean values show renal function declining with increasing age. “n”, number of measurements contributing to each boxplot.

For 52 patients, 195 values of eGFR could be calculated before KRT and assigned to CKD stages (Figure 1).

### 3.5. Kidney Replacement Therapy

Thirty-one patients (41.9%), 17 female and 14 male, underwent KRT (either dialysis or pre-emptive kidney transplantation), data was missing in four cases. The median age at initiation of KRT was 10 years (IQR: 9–15, range: 7–25).

Thirty patients underwent kidney transplantation at least once. 18 of those patients received their first transplant after a period of dialysis therapy, 4 kidneys were transplanted pre-emptively whereas in 8 cases data on preceding dialysis was missing. One patient on chronic dialysis therapy had not yet been listed for transplantation.

**Figure 2:**
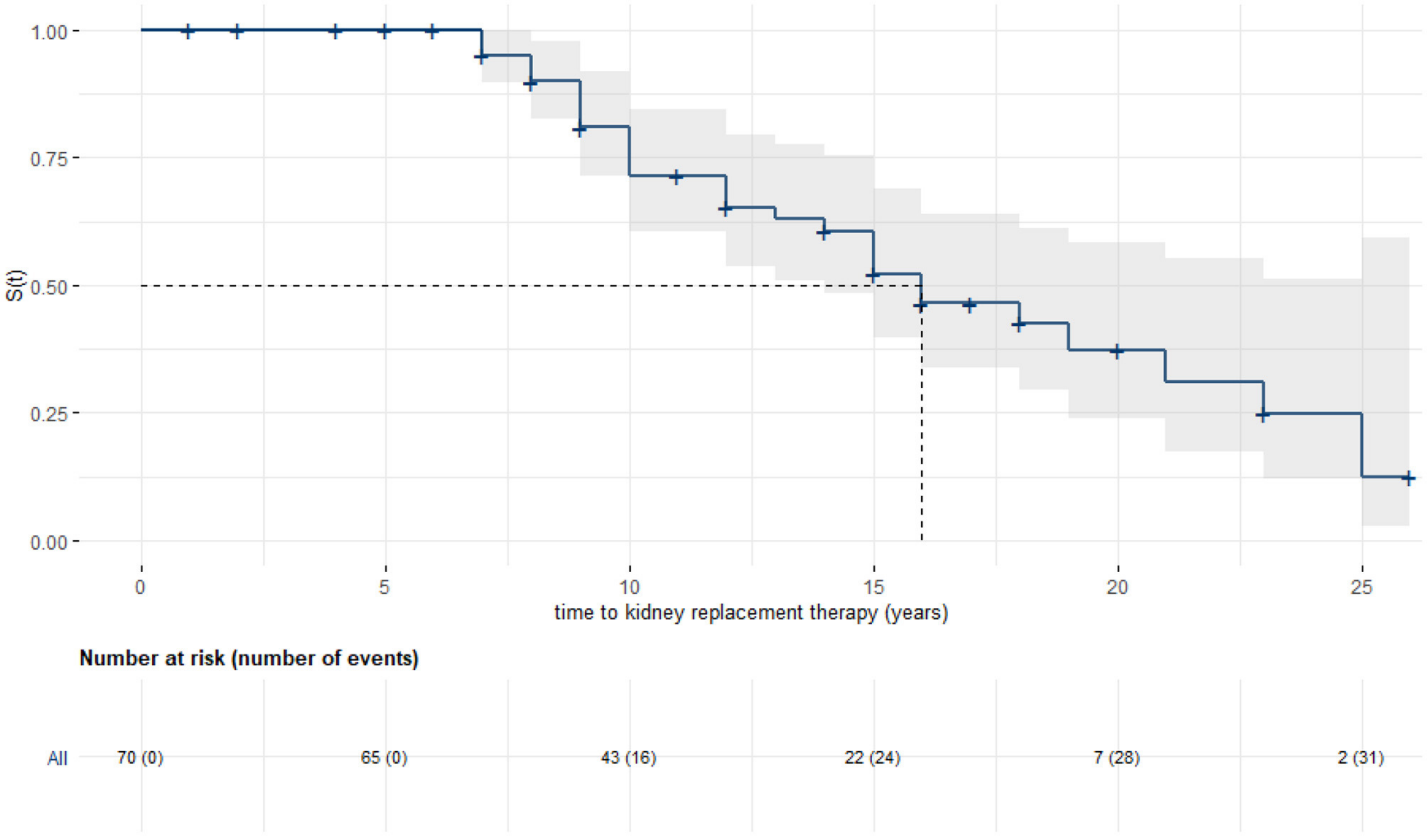
Kaplan–Meier analysis with 95% confidence band: Kidney survival in years. Seventy patients could be included, median renal survival was 16 years (95% CI: 14, -).

**Figure 3:**
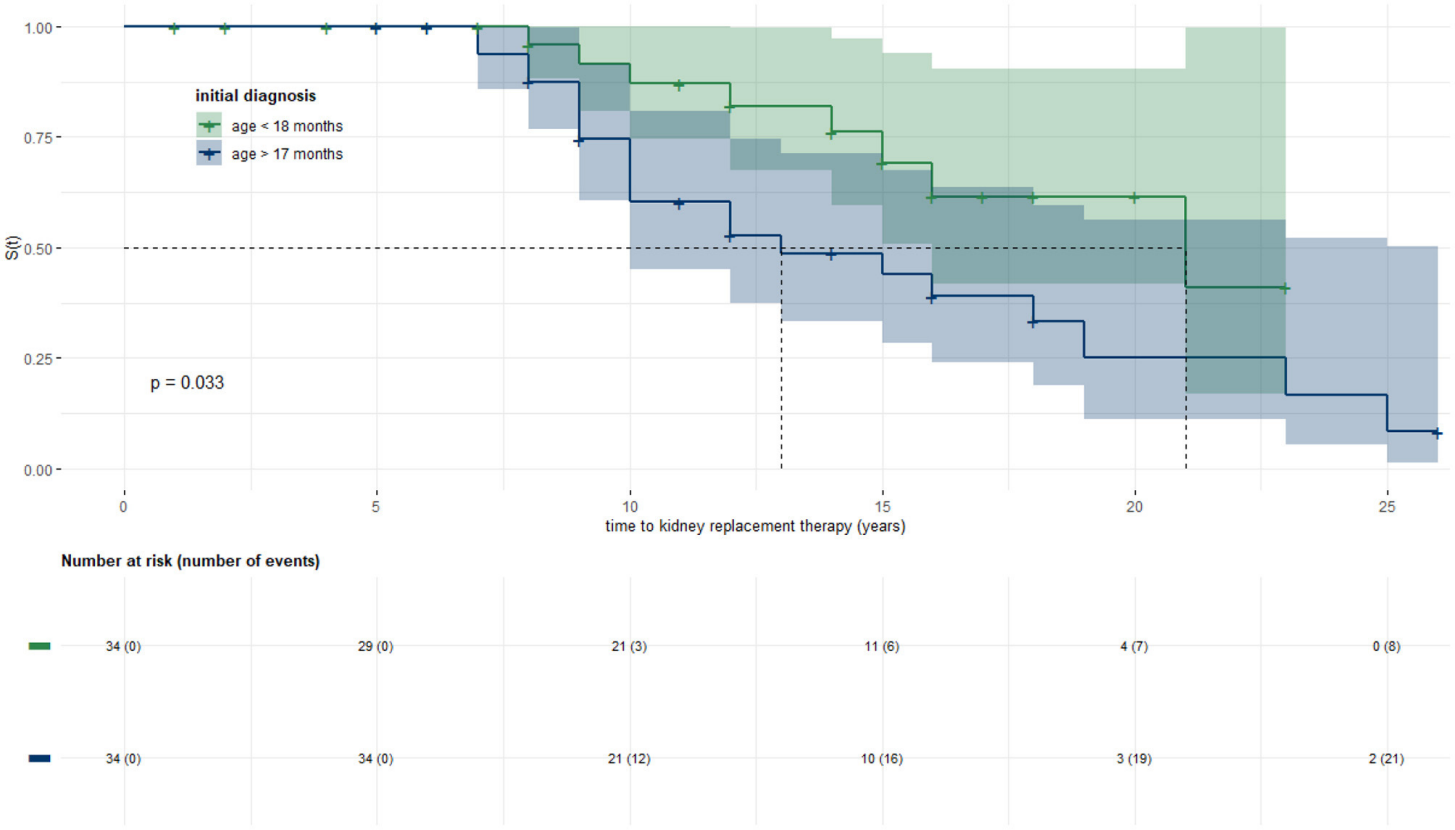
Stratified Kaplan–Meier analysis with 95% confidence bands and log-rank test: Kidney survival in years. Groups were stratified for age at initial diagnosis (cut-off point at 18 months). The 34 patients each showed a median renal survival of 13 years (95% CI: 10, -) in the group diagnosed at an older age vs. 21 years (95% CI: 16, -) in the group with earlier diagnosis.

Kaplan–Meier analysis was used to evaluate time from birth to KRT (Figure 2). Data was stratified for age at initial diagnosis in months (Figure 3). Thirty-four patients (45.9%) were each assigned to one of two groups (initial diagnosis before 18 months of life vs. at 18 months or older), for which a log-rank test showed a significant difference in renal survival.

When stratified for year of birth (1975 to 2001 vs. 2002 to 2019) or sex, with crossing hazards and therefore failing log-rank test, no difference in renal survival was detected.

### 3.6. Growth

**Figure 4:**
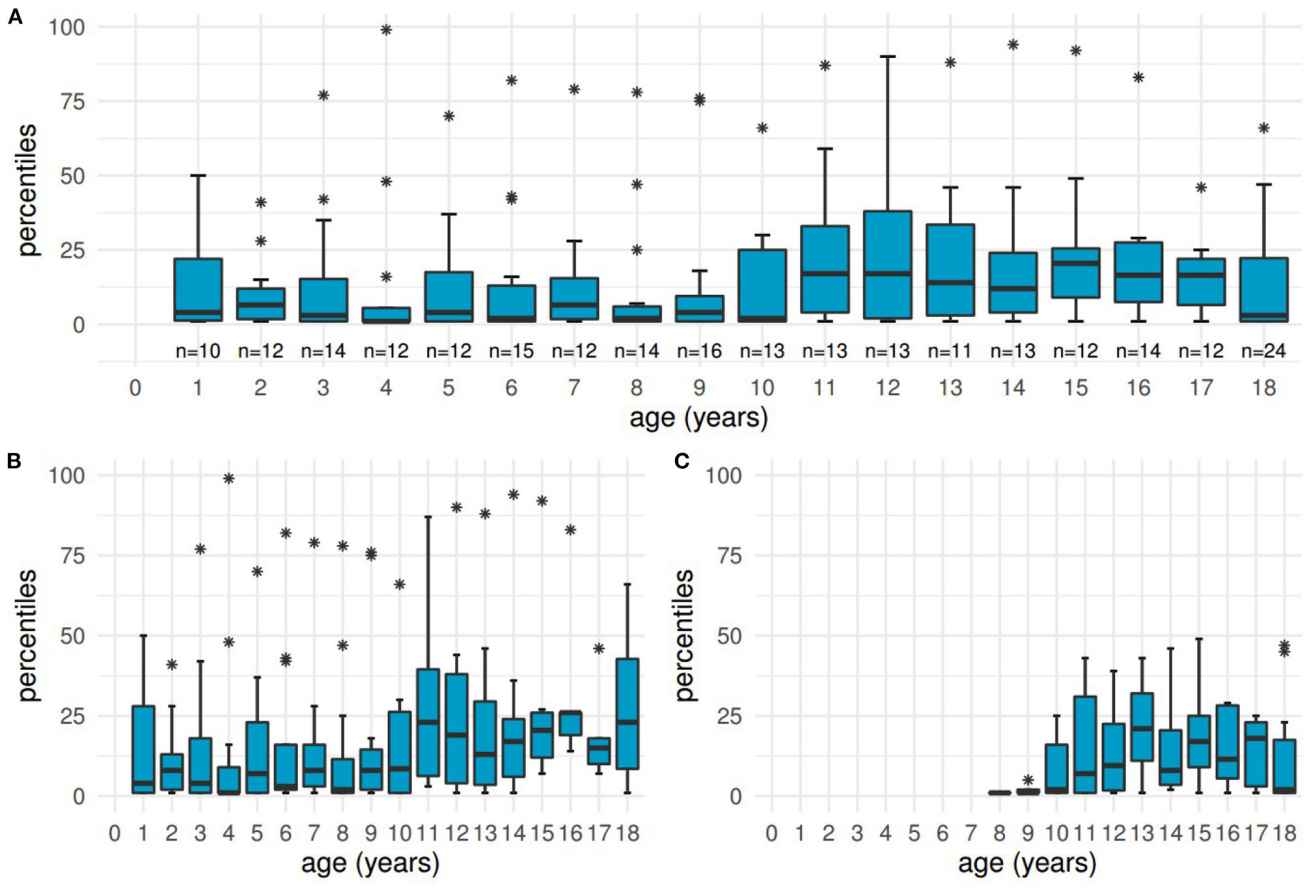
Distribution of height at different ages. Boxplots showing low percentiles at the ages of 1 year to adulthood, with the age of “18” including the heights of all adult patients. **(A)** all patients, **(B)** subgroup of 41 patients before initiation of kidney replacement therapy, **(C)** subgroup of 27 patients receiving kidney replacement therapy.

Percentiles for height were calculated for 68 patients with a total of 242 documented values available. The overall median lay at the 7^th^ percentile (IQR: 1–25, range: 1–99). Detailed boxplots are shown in Figure 4, displaying all age groups from 1 year to adulthood and differentiating two subgroups (before and after initiation of KRT).

Ninety-eight values (40.5%) from 43 patients were within the defined pathological range of ≤ 3^rd^ percentile at a median age of 8 years (IQR: 5–13, range: 1–18 and older). Pathological heights were mostly documented for younger children and adults born between 1975 and 2001. Only 3 patients reached the 50^th^ percentile or higher at least once.

The subgroup not yet in need of KRT (41 patients, 164 measurements) had a median percentile of 8 (IQR: 1–26, range: 1–99), the median of 27 patients after initiation of KRT lay at the 6^th^ percentile (IQR: 1–23, range: 1–49, including 66 values).

Percentiles for weight were calculated for 71 patients with a total of 248 documented values available. The overall median lay at percentile 15.50 (IQR: 5.00–34.25, range: 1.00–97.00). Fifty-three values (21.4%) from 28 patients were ≤ 3^rd^ percentile at a median age of 8 years (IQR: 4–13, range: 1–18 and older). Only 16 patients reached the 50^th^ percentile or higher at least once.

Data on tube feeding was available for 25 patients (33.8%). Median age when beginning tube feeding was 1 year (IQR: 1–3, range: 1–10). For 11 patients, the median duration was 6 years (IQR: 2–13, range: 0.04–23), whilst for 13 patients the median duration was at least 4 years (IQR: 2–5, range:1–11), and was still ongoing through the period of observation.

Thirty-nine (52.7%) of the patients received recombinant growth hormone therapy at some point. In 10 cases, the exact duration was known (mean: 7.70 years, SD: 3.53, range: 1.00–13.00); in those cases the therapy started at a mean age of 8.00 years (SD: 3.62, range: 1–12). In 26 cases, the documented duration was a median of at least 4.00 years (IQR: 1.25–7.50, range: 1.00–15.00), but the exact starting and/or ending points remained unclear due to insufficient documentation.

### 3.7. Extrarenal Manifestations

Observed extrarenal manifestations are listed in Table 2. The available documents did not distinguish the absence of those symptoms from missing information.

**Table 2:** Extrarenal manifestations.

**Manifestation**	**Counts**	**Percentage**
Anemia	29	39.2
Hypothyroidism	34	45.9
Skeletal deformities	51	68.9
Rickets	16	21.6
Ophthalmological symptoms	42	56.8
Neurological symptoms	11	14.9
Muscle weakness/hypotension	10	13.5

The most reported skeletal deformities were genu valgum (33 cases), pes planovalgus (10 cases), and scoliosis (7 cases). Where further details on ophthalmological symptoms were given, photophobia and corneal discomfort were reported to be the main concern. Regarding neurological symptoms, 7 patients with seizures were reported, and there was one case each of movement disorder, dyslalia, motor retardation and stroke with speech impediments. There were no entries on diabetes, but one patient was reported as suffering from exocrine pancreatic insufficiency. Detailed information on the context of these observations was not available.

Gastrointestinal symptoms are presented in Table 3.

**Table 3:** Gastrointestinal symptoms.

**Manifestation**	**Counts**	**Percentage**
Nausea	20	27.0
Vomiting	21	28.4
Dysphagea	10	13.5
Aches	8	10.8
Diarrhea	3	4.1
Obstipation	1	1.4
Bleeding	1	1.4
Unspecified	3	4.1

*No data available on 37 patients*.

## 4. Discussion

Our results indicate that cystine-depleting treatment of INC in Germany and Austria is comparable to other recently reported international data with the length of renal survival being determined by age at diagnosis and subsequent initiation of cysteamine therapy.

### 4.1. Clinical Presentation

Despite the tremendous progress treatment has made within the last decades, INC continues to be a severe chronic condition (16, 28, 29).

Frequently, the first sign of INC noticed by parents and clinicians is failure to thrive (38). We observed that children often needed the support of tube feeding at a young age, continuing for several years. Growth has been an important outcome parameter in previous studies as it reflects the multi-factorial influences of different aspects of the disease as well as therapeutic success (29, 38). We were able to show that even though medication in Germany and Austria started at an early age, patients remained very short in stature. Our results are in concordance with another study focusing on patient's body growth before initiation of KRT (23). However, our data was not sufficient to explain the great interindividual variety, with several patients having fewer difficulties than others. These results might indicate an improvement around length of therapy and medical developments in recent years, as pathological percentiles were mostly concentrated at younger ages and adults born before 2001. While thorough analyses of its impact on growth in INC patients are still necessary, we recommend an early start of GH treatment in infancy for children with INC based on the suggestion directing the general population of infants with CKD (39).

Our study also demonstrated that there was subsequent kidney failure with advancing age. Overall median renal survival in German and Austrian patients corresponded to the large European-Turkish cohort analyzed recently by Emma et al. (29). We also showed that in German-Austrian patients, time of initial diagnosis accounted for a great difference in median renal survival with our cut-off point at the age of 1.5 years (arbitrarily chosen at a younger age than in previous studies (15, 30), oriented to age at initial diagnosis in our cohort).

Information on extrarenal symptoms and their onset could not be determined as thoroughly as planned, but our results still provided an insight into the greatest challenges regarding this disease. Corresponding to international studies (30, 40), hypothyroidism, anemia, ophthalmological, skeletal and gastrointestinal complications were very common manifestations of INC. Considering the incomplete health records on this topic, it is likely that more patients were affected than was actually documented in the present study.

### 4.2. Quality of Healthcare

We aimed to characterize the clinical course for German-Austrian patients with INC, with a main focus on assessing the quality of their healthcare using data on initial diagnosis and treatment as well as undertaking an analysis of the dosage and monitoring of specific therapies.

According to the findings of Bertholet-Thomas et al. (15), our cohort was representative for management of INC in developed nations. We showed that systemic application of cysteamine was offered to patients very soon after initial diagnosis, addressing the importance of cystine-depleting therapy. Prescribed doses of systemic cysteamine were at an optimal level according to international standards (12), regardless of whether IRC or ERC was used. No relevant overdoses were documented.

As the measurement of LCLs provides an indirect insight into patients' adherence and overall success of cysteamine treatment, analyzing this aspect thoroughly was of importance in this study. The aim of the therapy is to remain ≤ 1 nmol cystine/mg protein, with an optimal outcome being a level ≤ 0.5 (31). We found our cohort to have overall satisfying LCLs when calculating the time-averaged mean for each patient as well as when considering all measurements individually. The therapy in our cohort seemed to be particularly efficient after a few years of treatment which is indicated by the observation that those values above 1 nmol cystine/mg protein are found at younger ages than the sufficient LCLs. The comparison of our findings with international data (15, 29) is remarkable as it shows that, though there may have been room for improvement on an individual level, this cohort had exceptional overall therapy control based upon LCL. However, the assay to detect LCL, the current gold standard to monitor cysteamine therapy, is technically demanding and not always available. Biomarkers of macrophage activation are promising monitoring candidates, reflecting the macrophage-mediated inflammation in cystinosis. Among them, chitotriosidase enzyme activity showed to be a good predictor of LCL and was significantly correlated to extrarenal complications (41). Concentration of ceroid in macrophages (a product of lysosomal oxidation of LDL that plays an important role in the development of atherosclerosis) might become a future marker to monitor the antioxidative properties of cysteamine (42). Monitoring of therapy adherence should not be restricted to the analysis of direct cysteamine effects, but should also include biomarker tracking of disease complications, such as those of bone status (43), thyroid function and glucose metabolism. The most relevant clinical biomarker correlated to cysteamine effect doubtlessly remains preservation of glomerular filtration rate (44) and might be the best motivation for therapy adherence.

Age at initial diagnosis in our patient cohort was comparable to recent experiences in other countries (15, 29). Interestingly, in our cohort spanning four decades, a more recent date of birth was not associated with earlier diagnosis or relevantly improved renal survival, leading to the hypothesis that the awareness for diagnosis of cystinosis in the first year of live has not increased and an optimal therapeutic approach might have already been reached. Thus, new initiatives, such as new-born screening to enable earlier diagnosis, need to be implemented to achieve even more favorable outcomes. It has to be noted, though, that it is currently unclear whether cysteamine treatment bears more advantages than risks when initiated before children experience symptoms of INC.

### 4.3. Limitations

Limitations of our study were closely linked to its retrospective nature, and mainly consist of incomplete records on some patients as well as heterogeneous distribution of duration of individual follow-up.

There was no information available on the underlying genetic defect.

Particularly in cases where there was no reporting on extrarenal manifestations, it remained unclear whether the information was missing or whether the symptoms did not occur, so more patients might have been affected. Our numbers on those manifestations are therefore to be understood as minimal counts. Also, patient ages at onset remained uncertain. Concerning all extrarenal manifestations and especially our data on growth, important information could not be evaluated: Data on supporting medication, nutritional intervention, acidosis, bone disease and other disease-related as well as therapeutic factors was not available.

For Kaplan–Meier-analyses, data on eight patients was included although it was not known whether these persons had received dialysis before their documented transplantation date. In our opinion, however, it was deemed important to include data on these patients nevertheless to avoid loss of valuable information.

Additionally, despite being a large cohort for national studies on this orphan disease, statistical analysis was still flawed because of its relatively small sample size.

A certain level of bias might have also played a role as patients' dates of birth varied greatly and health care has changed fundamentally for patients born more recently. Changes in awareness and profound knowledge of INC, pharmaceutical developments such as the availability of cysteamine and growth hormones, advances in KRT but also individual patients' characteristics such as adherence and psychosocial aspects in general may have had an impact on our observations that we cannot differentiate nor quantify.

Furthermore, there may have been sampling bias as there are rarely coordinated programs for transitioning care to specialized centers in adulthood and it was therefore very difficult to include adult patients in this study. Adult nephrologists were very widely contacted but did not report back. The majority of all pediatric nephrology centers in Germany that treat patients with cystinosis have included their data in the registry of the German Society for Pediatric Nephrology. In our view, establishing national and international registries will be the key to understanding and managing this orphan disease in the future.

## 5. Conclusion

This study is the first to extensively characterize a large cohort of patients with INC in Germany and Austria, with a focus on the previous decade. Our findings underline that continuous monitoring of the effects of treatment in rare diseases by international initiatives is needed. Our results confirm recent international reports that treatment with cysteamine is adequate, whereas other disease aspects, such as growth and treatment of extrarenal manifestations, should be optimized in the future.

## Data Availability Statement

The raw data supporting the conclusions of this article will be made available by the authors, without undue reservation.

## Ethics Statement

The studies involving human participants were reviewed and approved by Ethics Committee Hannover Medical School. Written informed consent to participate in this study was provided by the participants' legal guardian/next of kin.

## Author Contributions

LPe and JO designed the study. NO'C collected the data and performed the statistical analyses. LPe and NO'C wrote the first draft of the manuscript. LPe, JO, KA, AB, DH, JK, CK, CM, DM, LW, BT, MW, and KH provided patient data. All authors contributed to manuscript revision, read, and approved the submitted version.

## Funding

LPe received an unrestrictive grant from Chiesi Germany to conduct this study. The funder was not involved in the study design, collection, analysis, interpretation of data, the writing of this article or the decision to submit it for publication.

## Conflict of Interest

DH received speaker fees and research support, CK and BT received speaker and advisory board fees from Chiesi. LPe, LW, and JO received speaker fees from Chiesi and Orphan Europe.

The remaining authors declare that the research was conducted in the absence of any commercial or financial relationships that could be construed as a potential conflict of interest.

## Publisher's Note

All claims expressed in this article are solely those of the authors and do not necessarily represent those of their affiliated organizations, or those of the publisher, the editors and the reviewers. Any product that may be evaluated in this article, or claim that may be made by its manufacturer, is not guaranteed or endorsed by the publisher.

## Abbreviations

CKD: chronic kidney disease
95% CI: 95% confidence interval
eGFR: estimated glomerular filtration rate
ERC: extended-release cysteamine
IRC: immediate-release cysteamine
IQR: interquartile range
LCL: leukocyte cystine level
INC: infantile nephropathic cystinosis
KRT: kidney replacement therapy
SD: standard deviation.
