# Supplementary figures and images for: Patients With Infantile Nephropathic Cystinosis in Germany and Austria: A Retrospective Cohort Study
Source: Front Med (Lausanne). 2022 Apr 25;9:864554. doi: 10.3389/fmed.2022.864554 (PMC9082678; doi:10.3389/fmed.2022.864554)

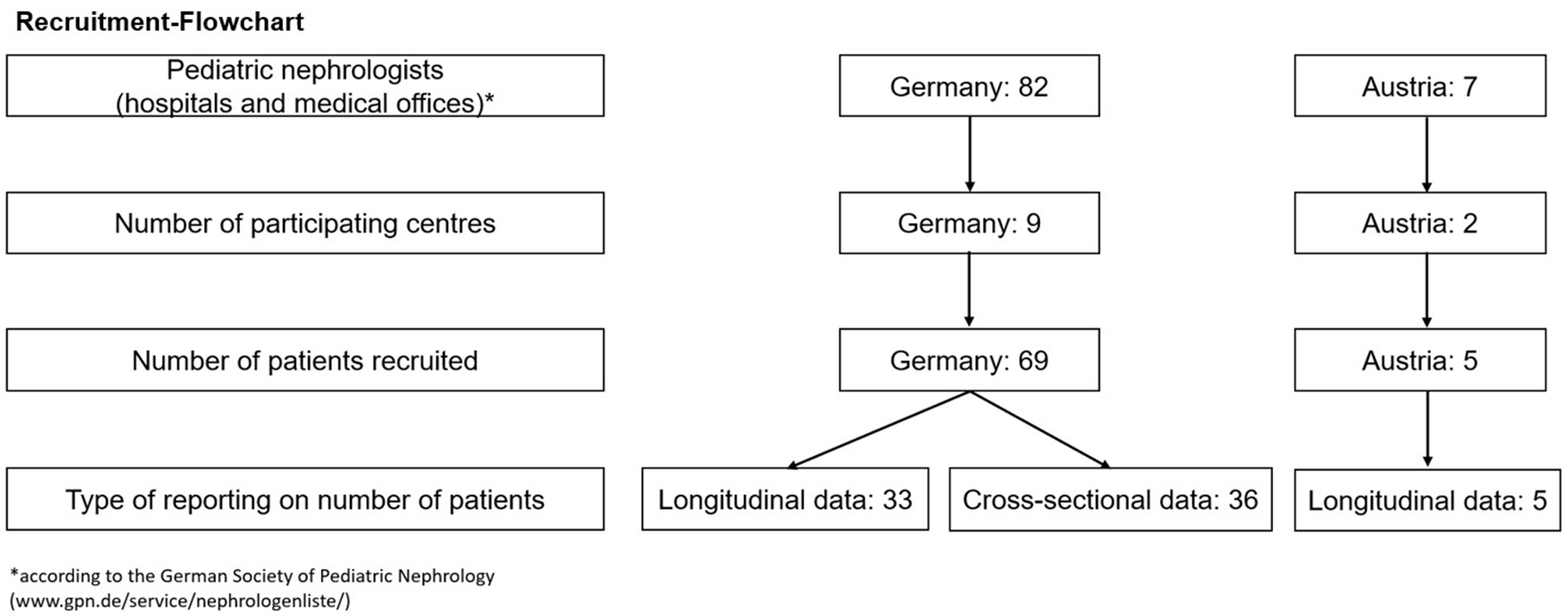

Supplement: Supplementary file 1 [file Image_1.JPEG]
